# Insecticide resistance in *Aedes aegypti *populations from Ceará, Brazil

**DOI:** 10.1186/1756-3305-4-5

**Published:** 2011-01-12

**Authors:** Estelita Pereira Lima, Marcelo Henrique Santos Paiva, Ana Paula de Araújo, Éllyda Vanessa Gomes da Silva, Ulisses Mariano da Silva, Lúcia Nogueira de Oliveira, Antonio Euzébio G Santana, Clarisse Nogueira Barbosa, Clovis C de Paiva Neto, Marilia OF Goulart, Craig Stephen Wilding, Constância Flávia Junqueira Ayres, Maria Alice V de Melo Santos

**Affiliations:** 1 Universidade Federal do Ceará - Avenida da Universidade, 2853, CEP 60020-181, Benfica, Fortaleza/CE, Brasil; 2 Centro de Pesquisas Aggeu Magalhães/Fiocruz - Avenida Professor Moraes Rego, s/n, CEP 50670-420, Campus UFPE, Cidade Universitária, Recife/PE, Brasil; 3 Faculdade ASCES - Avenida Portugal, 584, CEP 5016-400, Bairro Universitário, Caruaru/PE, Brasil; 4 Universidade Federal de Alagoas - Campus A. C. Simões - Avenida Lourival Melo Mota, s/n, CEP: 57072-970, Tabuleiro do Martins, Maceió/AL, Brasil; 5 Núcleo de Entomologia Médica do Cariri - Rua Manoel Couto Soares, 664, CEP 63180-000, Novo Juazeiro, Juazeiro do Norte/CE, Brasil; 6 Vector Group, Liverpool School of Tropical Medicine - Pembroke Place, Liverpool L3 5QA, UK

## Abstract

**Background:**

Organophosphates and pyrethroids are used widely in Brazil to control *Aedes aegypti*, the main vector of dengue viruses, under the auspices of the National Programme for Dengue Control. Resistance to these insecticides is widespread throughout Brazil. In Ceará the vector is present in 98% of districts and resistance to temephos has been reported previously. Here we measure resistance to temephos and the pyrethroid cypermethrin in three populations from Ceará and use biochemical and molecular assays to characterise resistance mechanisms.

**Results:**

Resistance to temephos varied widely across the three studied populations, with resistance ratios (RR_95_) of 7.2, 30 and 192.7 in Juazeiro do Norte, Barbalha and Crato respectively. The high levels of resistance detected in Barbalha and Crato (RR_95 _≥ 30) imply a reduction of temephos efficacy, and indeed in simulated field tests reduced effectiveness was observed for the Barbalha population. Two populations (Crato and Barbalha) were also resistant to cypermethrin, whilst Juazeiro do Norte showed only an altered susceptibility. The *Ile1011Met kdr *mutation was detected in all three populations and *Val1016Ile *in Crato and Juazeiro do Norte. *1011Met *was significantly associated with resistance to cypermethrin in the Crato population. Biochemical tests showed that only the activity of esterases and GSTs, among the tested detoxification enzymes, was altered in these populations when compared with the Rockefeller strain.

**Conclusions:**

Our results demonstrate that two *A. aegypti *populations from Ceará are under strong selection pressure by temephos, compromising the field effectiveness of this organophosphate. Our results also provide evidence that the process of reducing resistance to this larvicide in the field is difficult and slow and may require more than seven years for reversal. In addition, we show resistance to cypermethrin in two of the three populations studied, and for the first time the presence of the allele *1016Ile *in mosquito populations from northeastern Brazil. A significant association between *1011M*et and resistance was observed in one of the populations. Target-site mechanisms seem not to be implicated in temephos resistance, reinforcing the idea that for the studied populations, detoxification enzymes most likely play a major role in the resistance to this insecticide.

## Background

In Brazil, the wide distribution of *Aedes aegypti *- vector of the three circulating dengue virus serotypes (DENV-1, -2 and -3) - and the lack of a specific treatment or vaccine for dengue, are considered serious public health issues. This situation is exacerbated by the recent re-introduction of serotype 4 (DENV-4) in the State of Roraima, in the North of Brazil [1].

*Aedes aegypti *is present in all states and in most cities of Brazil. In Ceará State, this mosquito has been detected in approximately 98% of cities [2]. In the last 23 years dengue has been endemic in the state, with four major epidemic periods registered in 1987, 1994, 2001 and 2008. In the latter year, Ceará was the most affected Brazilian state with respect to dengue cases, with 44,244 registered cases - an incidence of 530.77 cases/100,000 inhabitants. In 2009, although case numbers were lower than in the previous year, the mortality rate for dengue hemorrhagic fever was 34.6% and among severe dengue cases was 45% [2], significantly higher than the average mortality elsewhere in Brazil [2].

For the past 13 years, insecticides of the organophosphate (OP) and pyrethroid (PY) classes have been utilized intensively as the main strategy to control *A. aegypti*, run by the National Programme for Dengue Control (Programa Nacional de Controle da Dengue - PNCD) [3,4]. Monitoring of the susceptibility status of *A. aegypti *populations to these insecticides has been undertaken through the National Network for Monitoring Resistance to Insecticides in *Aedes aegypti *(Rede Nacional de Monitoramento da Resistência de *Aedes aegypti *a Inseticidas - MoReNAa), since 1999 [5]. The first signs of incipient resistance to temephos were registered in 1999 in mosquito populations from the State of São Paulo [6], and in other states since 2001 [4]. Currently, it is known that temephos resistance is widespread in *A. aegypti *populations throughout the country [5,7-12]. Consequently, the MoReNAa network has also been working on assaying the activity of detoxification enzymes in *A. aegypti *populations since 2001, aiming at identifying alterations that may be related to temephos resistance [12,13].

In Ceará, resistance to temephos was detected in surveys conducted from 2000-2002 in *A. aegypti *populations from Fortaleza, Caucaia [14] and Juazeiro do Norte [15]. In an effort to manage resistance in the field, temephos was replaced by the biolarvicide *Bacillus thuringiensis israelensis *(Bti) in these cities, and others, as a recommendation of the MoReNAa network [5]. However, in most of the municipalities of Ceará (164) temephos continued to be utilized until the beginning of 2010, including Crato and Barbalha, where resistance to this insecticide had been first detected in *A. aegypti *in 2003 [9]. Although the dengue control program at city level prioritizes the use of insecticides, a continuous evaluation of the effectiveness of temephos-based products in the field is not performed in most of them [2], despite PNCD recommendations to do so.

Data released by the MoReNAa network obtained from sentinel cities, revealed that *A. aegypti *populations were also resistant to malathion (another OP used as an adulticide) in 1999/2000, when its use was interrupted and replaced by pyrethroids as a resistance management action [5,10]. However, this change led to cypermethrin/deltamethrin resistance three years later [16]. After detecting resistance to all insecticides used by PNCD, this network carried out studies to characterize resistance mechanisms in Brazilian field populations.

Generally, two main mechanisms are commonly associated with resistance to chemical insecticides: metabolic, via increased activity of detoxification enzymes such as esterases, mixed function oxidases (cytochrome P450s) and glutathione S-transferases (GSTs); and structural modifications in insecticide binding sites, such as acetylcholinesterase and the voltage-gated sodium (Na_V_) channel. Recent studies suggest that in addition to metabolic resistance, mutations in the sodium channel (the target of pyrethroids as well as DDT) may be playing a role in the resistance to cypermethrin and deltamethrin in mosquito populations from Brazil [17].

PY compounds act on the insect nervous system, targeting the Na_V _channel. This channel is composed of four domains (I-IV) and each domain comprises six transmembrane helices (S1-S6) [18,19]. Pesticides such as PY and DDT retard the activation and inactivation potential of Na_V _channels, triggering a series of repetitive discharges in motor and sensory axons, and resulting in paralysis ("knock-down") and death [20]. However, several insect species, including *Anopheles gambiae*, *Culex pipiens*, *Culex quinquefasciatus *and *A. aegypti*, may present a resistance phenotype to chemicals which target the Na_V_, commonly called knock-down resistance [17,21-27]. The genetic basis of knock-down resistance was first elucidated in the housefly *Musca domestica *[28]. The knock-down resistance trait (named *kdr*) and another *kdr*-related trait (super-*kdr*), which confers greatly elevated resistance in combination with *kdr*, were mapped to chromosome 3 [19]. Both traits have been associated with a lower electrophysiological sensitivity of elements from the nervous system and a reduced function of the Na_V _channel. Many studies have focused on finding mutations in Na_V _channel sequences from knock-down resistant populations. Characterization of sequences from *A. gambiae *and *C. quinquefasciatus *pyrethroid-resistant strains showed that the most common mutation is a leucine to phenylalanine substitution in the S6 hydrophobic segment of domain II [21,22], although a leucine to serine mutation has also been reported at the same 1014 site [24,29]. Brengues *et al*. [30] have failed to find the same mutation in *A. aegypti*, however, other studies have found different mutations correlated with *kdr *in this vector, such as: *Gly923Val*, *Leu982Trp*, *Ile1011Val*, *Ile1011Met*, *Val1016Ile *and *Val1016Gly *[17,25,30]. A few reports have shown that *kdr *genotyping is a good predictor of susceptibility to pyrethroid and DDT, and, at the moment, it is considered the best tool for predicting the efficacy of these compounds in the field [31].

The aim of this study was to measure resistance levels to two insecticides and to characterize resistance mechanisms at both the molecular and biochemical levels in *A. aegypti *populations from Ceará State.

## Methods

### Characterization of study area

**Figure 1:**
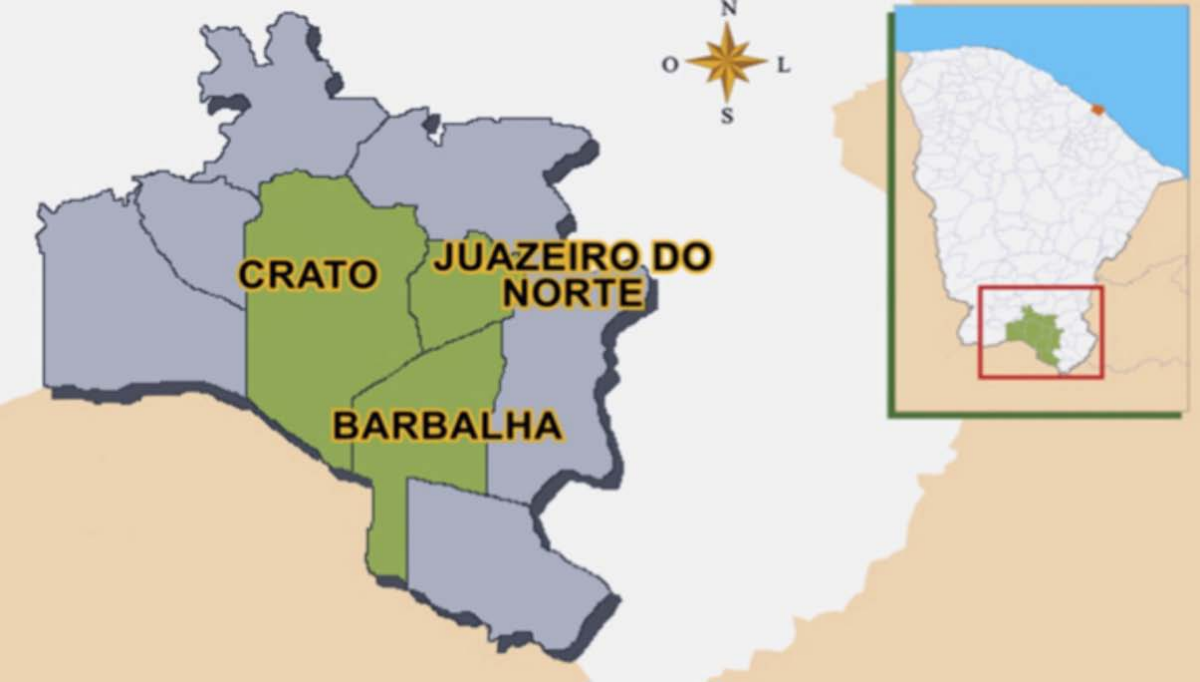
Map of Ceará State, Brazil, and in detail the three localities studied.

*mosquito populations *- this study was undertaken in three municipalities, Juazeiro do Norte, Crato and Barbalha, located in the south region of Ceará State, Brazil, (Figure 1), 10 Km equidistant from each other. The local climate is tropical semi-arid, with average temperatures of 24-26°C and rainfall of 925 to 1,156 mm per year [32].

Juazeiro do Norte (7° 12' 47" S, 39° 18' 55" W) is 248.55 Km^2 ^in area, and has a population of 249,831 with 50,000 properties [33]. This city is the main commercial centre of the south region and is also one of the main religious routes of Brazil, attracting thousands of visitors each year from throughout the country, but particularly from the Northeast region. Crato (7° 14' 03" S, 39° 24' 34" W) has an area of 1,009.20 km^2^, 116,759 residents and 20,185 properties. This city is on the slope of the Araripe plateau. Barbalha (7° 18' 40" S, 39° 18' 15" W) has an area of 479.184 km², 52,496 residents and 7,032 properties.

Mosquito samples were collected in the field in November and December 2009 with the use of 100 oviposition traps (ovitraps), adapted from [34], distributed homogeneously in each area, based on the recommendations of the MoReNAa network [12,35]. More than 50% of these ovitraps were positive for *Aedes *spp eggs. Field derived colonies from each selected population were established with approximately 2,000 mosquitoes (males and females) from these eggs. Populations were kept under controlled conditions in the insectary of the Federal University of Ceará (Cariri Campus), in order to obtain filial generation (F1), which was utilized in the biological, biochemical and molecular experiments described below. The Rockefeller strain, the standard strain for insecticide susceptibility tests, and used in all experiments as a susceptible reference strain, was obtained from a sub-strain kindly provided by Núcleo de Pesquisa/Sucen/Marília-SP (member of the MoReNAa/MS network, and initially provided by CDC, Puerto Rico).

### Insecticides

temephos, an organophosphate larvicide, was used under two formulations: a standard powder (Pestanal, 97.5%, Sigma lot 6333X), used to make a stock solution in ethanol for *in vivo *laboratory bioassays, and as sand granules with 1% active ingredient (Fersol, lot 197), utilized in the simulated field trials. The pyrethroid adulticide cypermethrin, as a standard powder (98%, Sigma lot 127K1099), was dissolved in acetone for the impregnated bottles assay.

### In vivo bioassays with larvae

Dose-response bioassays were undertaken according to the methodology proposed by the World Health Organization to evaluate larval susceptibility to temephos [36]. In these experiments, third-instar larvae (L3) were exposed to 10 different concentrations of the larvicide determined according to previous exploratory bioassays. For each concentration, and for the control, three replicates of 20 larvae were tested. Larval mortality was checked 24 h after exposure. All tests were repeated at least three times. Mortality data were utilized to calculate the lethal concentrations for 95% of exposed individuals (LC_95_) through linear regression log probit analysis, using SPSS software (version 8.0 for Windows). These values were compared to those obtained for the Rockefeller strain in order to estimate the resistance ratio (RR) for each population. The following criteria proposed by Mazzari and Georghiou [37] was adopted to classify the resistance level of populations: low (RR < 5), moderate (RR > 5 < 10) or high (RR >10).

### Simulated field trial

the aim of this trial was to verify the effectiveness of temephos in its sand granules formulation to control larvae in the field presenting distinct resistance levels, using the concentration recommended by the PNCD (1 ppm). The trials were performed in the external area of the Department of Entomology of the School of Medicine in Cariri/Barbalha, based on recommendations by the MoReNAa network [12]. Plastic reservoirs filled with 20 L of tap water, treated with 2 g of temephos sand granules and colonized individually with 20 L3 larvae, were placed in a covered area protected from the sun and rain. One third of the water in the containers was renewed three times per week. For the Rockefeller and each field population, one control and three treated replicates were included. Mortality was verified 24 h after insecticide exposure and live larvae were removed before the weekly re-colonization of containers. The evaluation was continued for 60 days, a period of time similar to the interval between insecticide applications in the field performed by the national program. In these tests, only populations from Barbalha and Juazeiro do Norte were used, since samples from Crato were scarce.

### In vivo bioassays with adults

For each population, three tests were performed using bottles impregnated with insecticides, according to the methodology described by Brogdon and McAllister [38], and Melo-Santos [11]. For these, around 130 unfed one day-old females were utilized. Mosquitoes were exposed to a diagnostic dose of 8 μg/bottle of cypermethrin. Mortality was checked every 15 minutes over a period of 2 h, based on a lethal time to kill 100% of the population (LT_100_) of 15 mins at this concentration for the susceptible Rockefeller strain. All live and dead (those that did not move throughout the observation period) mosquitoes were transferred to another container free of insecticides and observed 24 h later, to determine the final mortality rate and the resistance status of populations. According to [39], populations were classified as susceptible when mortality was > 98%, in need of further evaluation when > 80% < 98%, or resistant when < 80%. Differences between resistant and susceptible individuals were evaluated among populations through Fisher's exact test. Following bioassays, females were stored according to phenotype, at -80°C for analysis of *kdr *mutations, which could possibly be associated with pyrethroid resistance.

### *Kdr *screening

Individual mosquitoes were separated according to the resistance phenotype, established at the final mortality (24 h) as resistant (survivors) and susceptible (dead mosquitoes) and homogenized in DNAzol^® ^(Invitrogen, Carlsbad, CA, USA), for DNA extraction according to the manufacturer's instructions. DNA samples were later processed in order to analyze *Ile1011Met *and *Val1016Ile Aa*Na_V _mutations. To detect the *Aa*Na_V _*Ile1011Met *mutation, primers were designed based on the IIS6 region, as described by Saavedra-Rodriguez et al [25] and Martins et al [17,25]. PCR reactions were performed using 0.2 units of *Pfu *polymerase (Promega), 0.5 μM dNTPs, 2 μM of each primer, 1.5 μM MgCl_2 _and 10 ng of DNA. PCR conditions were: 5 min at 94°C, followed by 30 cycles of 1 min at 94°C, 1 min at 58°C and 2 min at 72°C, with a final extension at 72°C for 10 min. PCR products were purified using GFX™ PCR DNA and Gel Band Purification Kit (Amersham Pharmacia Biotech) and sequenced (ABI 3000, Applied Biosystems). Sequences were manually edited. Detection of *Aa*Na_V _*Val1016Ile *mutation was carried out using the following allele-specific primers, designed by Saavedra-Rodriguez *et al *[25]: reverse primer (5'-GGATGAACCGAAATTGGACAAAAGC-3') and allele-specific forward primers (Val 5'-GCGGGCAGGGCGGCGGGGGCGGGGCCACAAATTGTTTCCCACCCGCACCGG-3'and Ile 5'-GCGGGCACAATTGTTTCCCACCCGCACTGA-3). PCR reactions and conditions were identical to the ones mentioned above. PCR products were run in 5% high-resolution agarose (Sigma-Aldrich) gels to determine genotypes (Val - 78 bp and Ile - 98 bp). To test for association between *kdr *mutations and resistance we used Haploview v4 [40].

### Biochemical assays

approximately 100 females, randomly sampled from each field populations, were assayed by biochemical tests in order to verify the activity of the following insecticide detoxification enzymes: esterases (evaluated through the use of α and β naphthyl for α-Est and β-Est, respectively, and p-nitrophenyl PNPA), mixed function oxidases (MFO), glutathione S-transferases (GSTs), and acetylcholinesterase (AChE; the target of temephos). Microplate assays were performed on one-day-old adult females kept at -80°C [12]. The Rockefeller strain was used as the susceptible reference. The proportion of individuals from each population presenting enzyme activity higher than that observed for the 99 percentile of Rockefeller strain was used for status classification as follows: unaltered <15%, altered >15% <50%, and highly altered >50% (for more details see [12,41]).

## Results

### Status of susceptibility to temephos

all tested populations presented some resistance level to temephos, with individuals from Crato and Barbalha presenting the highest levels and Juazeiro do Norte a moderate level (LC_95 _= 3.279 mg/ml, 0.510 mg/ml and 0.123 mg/ml respectively. c.f. 0.017 mg/ml for Rockefeller) (Table 1). Temephos has been used extensively over the period 2002-2009 in both Crato and Barbalha and in these municipalities resistance ratios have increased over this period with, for example, an increase in RR to temephos from 9.0 to 192.7 in Crato (21 fold increase) and from 7.5 to 30 in Barbalha (4 fold increase). In Juazeiro do Norte where temephos was substituted by Bti for larval control the RR to temephos decreased by 30% over this period (10.4 to 7.2) (Table 2).

**Table 1:** Lethal concentration for 95% of individuals (LC_9__5_) and resistance ratio (RR) to temephos, estimated for *Aedes aegypti *populations from Ceará State.

Populations	Number of larvae	**LC**_**95 **_**(mg/L)** (Confidence interval 95%)	Resistance ratio **(RR**_**95**_**)**
Crato	960	3.279 (1.316-11.429)	192.7
Barbalha	1380	0.510 (0.277-0.893)	30.0
Juazeiro do Norte	960	0.123 (0.094-0.177)	7.2
Rockefeller*	1440	0.017 (0.014-0.018)	1.0

*Control strain

**Table 2:** Amount of temephos sand granules (1%) utilized in Ceará for controlling *Aedes aegypti *larvae.

			Resistance ratio to **temephos (RR**_**95**_**)**	
			
Populations	Number of houses treated with temephos (2002 to 2009)	Use of temephos from 2002 to 2009 (Kg)	(2003)**	(2009)
Crato	1059.507	60352.7	9.0	192.7
Barbalha	447.043	29219.0	7.5	30.0
Juazeiro do Norte	2093.937	0*	10.4	7.2

* In this period temephos was substituted by *Bacillus thuringienis israelensis *(Bti).

** Resistance ratio evaluated by Lima et al., (2006).

### Effectiveness of temephos in the field

in the simulated field trial, temephos killed 100% of the larvae in the two tested populations up to the 4^th ^week after treatment. The larvicidal effect on the Juazeiro do Norte population varied between 90% and 98% from the 4^th ^to the 8^th ^week, while the mortality of larvae from the Barbalha population decreased to 66.6% in the 5^th ^week, indicating a lower mortality rate than that recommended for validating the residual effect of temephos, according to the MoReNAa network. As expected, the insecticide killed all larvae of the control group (Rockefeller) throughout the experiment.

### Status of susceptibility to cypermethrin

two of the three tested populations, Crato and Barbalha, were resistant to cypermethrin, with both populations presenting average mortality rates lower than 80% after 15 min and 24 h (Table 3). Crato was considered the most resistant population to this insecticide, since 51% of the exposed mosquitoes recovered 24 h after being removed to an insecticide-free recovery bottle. The mortality rate of individuals from Juazeiro do Norte indicated reduced susceptibility to this pyrethroid (Table 3). The differences in phenotypic distribution among populations were significant (*p *= 1.054 × 10^-11^).

**Table 3:** Mortality rate and susceptibility status of *Aedes aegypti *populations from Ceará-Brazil to the pyrethroid cypermethrin.

		Mortality rate (%) after cypermethrin exposure (8 μg/bottle)						
			
		Exposure time					Time for recovery	
			
Populations	Number of females	15 min	30 min	60 min	90 min	2 hours	24 hours	Susceptibility Status
Crato	139	70.6	90.3	97.3	99.0	100	49.9	Resistant
Barbalha	148	62.6	87.2	93.3	95.8	99.3	65.8	Resistant
Juazeiro do Norte	163	88.7	92.0	99.0	99.0	100	97.0	Verification required
Rockefeller*	138	100	-	-	-	-	100	Susceptible

*Control strain

### *Kdr *screening

**Figure 2:**
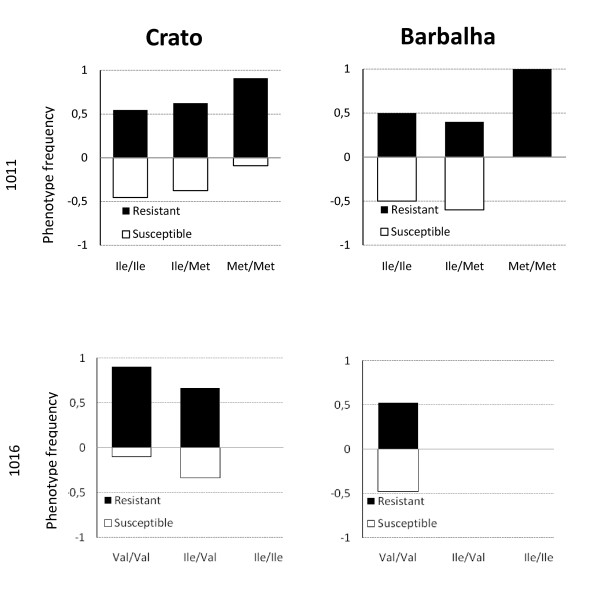
***Kdr *genotypes (1011 and 1016) and associated phenotype frequencies in *Aedes aegypti *from Ceará**.

Sequencing results showed that the *Ile1011Met kdr *mutation of the Na_v _channel was found in all three populations. The *1016Ile *allele was detected in Crato and Juazeiro do Norte (in heterozygotes only) but not in Barbalha. Significant differences were observed in genotype frequencies between populations: for *kdr1011*; Crato vs. Barbalha (*p *= 0.0218), and for *kdr1016*; Crato vs. Juazeiro do Norte (*p *= 3.81 × 10^-5^) and Crato vs. Barbalha (7.0844 × 10^-7^). Phenotype frequencies of each population classified by genotype are shown in Figure 2 and Tables 4 and 5.

**Table 4:** Genotype and allele frequencies of *Aedes aegypti kdr *mutation 1011 in pyrethroid susceptible and resistant individuals from Ceará.

		Genotype					Allele frequency	
		
Populations	Phenotype	Ile/Ile	Ile/Met	Met/Met	Total	N	Ile	Met
Crato	S	5 (0.56)	3 (0.33)	1 (0.11)	9	30	0.73	0.27
	R	6 (0.29)	5 (0.24)	10 (0.48)	21		0.41	0.59
Barbalha	S	4 (0.40)	6 (0.60)	0 (0.00)	10	21	0.70	0.30
	R	4 (0.36)	4 (0.36)	3 (0.28)	11		0.54	0.46
Juazeiro do Norte	S	20 (0.54)	11 (0.30)	6 (0.16)	37	37	0.69	0.31
	R	0	0	0	0		0	0

N = number of females; S = susceptible; R = resistant; Ile: isoleucine; Met: methionine

Barbalha and Juazeiro do Norte populations were found to be in Hardy-Weinberg equilibrium. However, the Crato population showed a significant deviation from Hardy-Weinberg expectation, due to a heterozygote deficit for *Ile1011Met *(*p *= 0.0197), and heterozygote excess for *Val1016Ile *(*p *= 0.015). The frequency of the *1011Met *allele was higher in resistant individuals from Crato, and significantly associated with resistance to cypermethrin (*p *= 0.0242). In Barbalha and Juazeiro do Norte, the resistance did not show any association with this allele (Table 4). Although the resistant allele *1016Ile *was observed in both resistant and susceptible *A. aegypti *from Crato, no association was found between this allele and the resistance status. For *kdr*1016 in samples from Barbalha all individuals were homozygous for the *1011Val *allele, thus, no association test was calculated. Since all individuals from Juazeiro do Norte were all phenotyped as susceptible, no test was performed for either putative *kdr *mutation (Table 5).

**Table 5:** Genotype and allele frequencies of *Aedes aegypti kdr *mutation 1016 in pyrethroid susceptible and resistant individuals from Ceará.

		Genotype					Allele frequency	
		
Populations	Phenotype	Val/Val	Val/Ile	Ile/Ile	Total	N	Val	Ile
Crato	S	1 (0.11)	8 (0.89)	0 (0.0)	9	30	0.56	0.44
	R	9 (0.43)	12 (0.57)	0 (0.0)	21		0.72	0.28
Barbalha	S	10 (1)	0 (0.0)	0 (0.0)	10	21	1	0
	R	11 (1)	0 (0.0)	0 (0.0)	11		1	0
Juazeiro do Norte	S	31 (0.84)	6 (0.16)	0 (0.0)	37	37	0.92	0.08
	R	0 (0.0)	0 (0.0)	0 (0.0)	0		0	0

N = number of females; S = susceptible; R = resistant; Val: valine; Ile: isoleucine

### Biochemical assays

**Figure 3:**
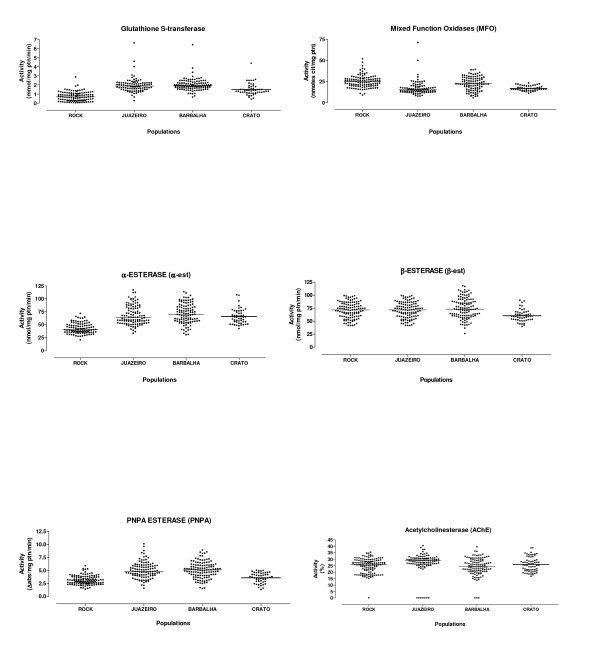
Profiles of enzyme activity in *Aedes aegypti *populations resistant to temephos, from Ceará State.

these assays showed an alteration in the activity of GST and α-esterase in all populations, in particular in individuals from Barbalha (Table 6, Figure 3). Esterases that metabolize PNPA were also altered only in the Barbalha and Juazeiro do Norte populations. Activity of other enzymes was not significantly different between populations when compared to the Rockefeller, including acetylcholinesterase (Table 6).

**Table 6:** Enzyme activity observed in *Aedes aegypti *from three insecticide resistant populations and the Rockefeller strain.

	α-esterase (nmol/mg ptn/min)			β-esterase (nmol/mg ptn/min)			Esterases-PNPA (Δabs/mg ptn/min)		
	
Populations/enzyme activity	N	Median	**99**^**th **^**Percentile**	N	Median	**99**^**th **^**Percentile**	N	Median	**99**^**th **^**Percentile**
Rockefeller	104	40.87	65.87	112	71.46	98.83	119	2.96	5.33

**Populations/enzyme activity**	**N**	**Median**	**(% > p99)**	**N**	**Median**	**(% > p99)**	**N**	**Median**	**(% > p99)**

Crato	51	64.58	43.0	51	59.42	0	51	3.57	0
Barbalha	108	70.03	57.0	116	73.12	13.0	119	5.11	41.0
Juazeiro do Norte^1^	108	63.67	45.0	89	74.72	7.0	108	4.83	39.0

**Populations/enzyme activity**	**Acetylcholinesterase (ACE)** **(% act) **^**a**^			**Glutathione S-transferase (GST)** **(mmol/mg ptn/min)**			**Mixed function oxidases **(**MFO)** **(nmol/mg ptn/min)**		
	
	**N**	**Median**	**99**^**th **^**Percentile**	**N**	**Median**	**99**^**th **^**Percentile**	**N**	**Median**	**99**^**th **^**Percentile**

Rockefeller	131	26.06	34.76	114	0.70	1.97	113	24.85	47.35

**Populations/enzyme activity**	**N**	**Median**	**(% > p99)**	**N**	**Median**	**(% > p99)**	**N**	**Median**	**(% > p99)**

Crato	65	25.30	6.0	51	1.39	18.0	50	16.23	0
Barbalha	113	24.17	3.0	116	1.90	42.0	101	22.54	0
Juazeiro do Norte^1^	109	28.90	5.0	98	1.89	42.0	92	15.32	2.0

N = number of females; % > p99: rate of the population with activity higher than the Rockefeller; (% > p99) < 15: unaltered enzyme activity; (% > p99) > 15 < 50 altered enzymatic activity; (% > p99) > 50: highly altered; ^a ^rate of activity in the presence of propoxur; ^1 ^population with reduced susceptibility to cypermethrin.

## Discussion

Over the last 10 years, resistance to organophosphate and pyrethroid insecticides employed for control of the dengue vector *Aedes aegypti *has been detected in all Brazilian regions, including Rio de Janeiro [4,12], Espírito Santo [35] and São Paulo [7,9-12] representing the Southeast, in all states from the Northeast region, in Distrito Federal and Goiás representing the Midwest region, and in Pará and Amazonas representing the North [8]. Here we now show that two populations of *A. aegypti *from Crato and Barbalha (Ceará State, Northeast Brazil) are resistant to both temephos and cypermethrin. The temephos resistance level of the *A. aegypti *population from Crato (RR = 192.7) was the second highest ever recorded in Brazil, surpassed only by Araripina, Pernambuco, Northeast Brazil (RR = 240) [11].

Over recent years resistance to temephos has risen markedly in Crato (2003 RR = 9.0; 2009 RR = 192.7) and Barbalha (2003 RR = 7.5; 2009 RR = 30). In this study the resistance level of a neighbouring population (Juazeiro do Norte) was much lower (RR = 7.2), which is only marginally below the 2003 level (RR = 10.2). Here, temephos has not been used for *Aedes *control for at least seven years, having been substituted by Bti. The increased resistance levels to temephos observed for populations from Crato and Barbalha, compared to Juazeiro do Norte where increase in resistance has been halted, indicate that the resistance management strategy utilized in the field in Juazeiro do Norte should have been extended to these neighboring cities. Our results also demonstrate that even though temephos has been substituted in Juazeiro do Norte, recovery of susceptibility has been slow. Melo-Santos *et al*. [11], in a study that simulated different field situations for reversion of temephos resistance in *A. aegypti*, suggested that besides the probable fitness cost associated with resistance, interruption of the selective pressure alone is insufficient to completely revert resistance and may prolong the state of intermediate or low resistance unless susceptible individuals are introduced. However, this species has a low dispersal rate in densely populated environments [42] suggesting gene flow between *A. aegypti *populations is low, despite their close proximity, as demonstrated previously by Ayres *et al*. [43] in their study of genetic variability of populations from Northeast Brazil.

The high resistance levels detected for Crato and Barbalha suggest that temephos will have low efficacy for *Aedes *control. Through simulated field trials we have confirmed the low effectiveness of temephos-based products, used at the field dosage, for the Barbalha population (RR = 30), corroborating Montella *et al*. [12], who showed that an RR > 10 can compromise insecticide effectiveness in control programs. Our results show clearly that the period for temephos larvicidal effectiveness (30 days) was much lower than the interval (60 days) recommended by PNCD between temephos application cycles in *A. aegypti *breeding sites. Consequently, there are serious faults in the vector control activities as currently undertaken in Barbalha and, probably in Crato (where RR = 192.7), although we did not undertake simulated field-trial evaluation of this population. Recent entomological indexes based on larval surveys conducted in these cities provide further evidence that the control program has not been successful in maintaining mosquito populations at low densities, since *A. aegypti *was detected in over 1% of houses [2]. The continued use of temephos in such areas will certainly decrease the efficacy of the insecticide within the scope of PNCD.

Resistance to temephos can arise through alterations in the target site of the insecticide (the acetylcholinesterase gene *ACE-1*) or through elevated levels or differential efficacy of metabolic genes. We utilized biochemical assays to characterize the mechanisms involved in insecticide resistance. No evidence was found for target-site resistance (AChE test). However, increased activity levels of GSTs, α-esterases and PNPA-esterases were detected indicating that members of these enzyme classes may play roles in detoxification and deserve further attention. Interestingly, *A. aegypti *from Crato, which presented the highest RR to temephos, has the lowest activity profile for these enzyme classes, indicating that the association between the increase of activity and level of temephos resistance is not simple. Thus, metabolic mechanisms do not seem to explain fully the elevated resistance levels to temephos, unless very few genes are involved whose activities could not be detected by our broad biochemical assays or unless other mechanisms, such as reduced insecticide penetration, are involved in temephos resistance. Future studies with synergists are warranted in order to provide additional information on metabolic mediated resistance mechanisms.

In Brazil, pyrethroids were first utilized by PNCD in 2000 [3,17], when resistance to temephos was spreading throughout the country [15]. Surveillance of cypermethrin susceptibility in *A. aegypti *populations from Brazil has been performed since 2001 when da Cunha *et al*. [16] demonstrated that populations from seven out of sixteen Brazilian cities presented an incipient alteration to this insecticide. Resistance increased over the following two years with 80% of the analyzed populations classified as resistant in 2003 [16]. Our results demonstrate that *A. aegypti *populations from Juazeiro do Norte present only a slight alteration in cypermethrin susceptibility, whilst for the Crato and Barbalha populations the mortality rates to PY is among the lowest registered for Brazilian populations [10,16]. Cypermethrin, alpha-cypermethrin or deltamethrin are sprayed monthly for mosquito control in strategic points (tire repair centres, cemeteries and junkyards). Additionally, ultra-low volume (ULV) spraying is recommended by the PNCD to block viral transmission as a complementary strategy when a high number of dengue cases are reported in specific areas [44] or in places with high mosquito infestation levels (>5%) in order to prevent outbreaks. In Juazeiro do Norte, the registered number of dengue cases was lower than Crato and Barbalha in the last year, implying that this population should have been subject to a lower insecticide pressure. In addition, since temephos is no longer used in this location, potential pyrethroid cross-resistance mechanisms would be minimized. As seen in other studies, resistance to PY seems to be rapidly evolving in populations from Ceará, where this insecticide started being utilized less than 10 years ago [10,16,45]. Additional factors that may have contributed to this rapid rise in resistance are: 1) the mechanisms involved in temephos resistance are also involved in PY resistance [46,47]; 2) a concomitant use of PY insecticides in other control programs, such as those for Leishmaniasis, malaria and Chagas disease; 3) large-scale PY usage to control urban pests (e.g. cockroaches, mites, ants and scorpions), performed by private companies acting independently of, and unsupervised by, the Secretaries of Health and Environment; and 4) domestic use of spray insecticides.

In recent years, various studies have shown an association between mutations in the voltage-gated sodium (Nav) channel and resistance to pyrethroids in various mosquito species [21,24,30,48-51]. In *A. aegypti*, a number of Nav mutations in segment 6 of domain II have been associated with *kdr*-like resistance, such as *Gly923Val*, *Leu982Trp*, *Ile1011Met*, *Ile1011Val*, *Val1016Ile *and *Val1016Gly *[17,25,30,52], *Asp1794Tyr *between segments 5 and 6 of domain II [53], and, more recently, *Phe1534Cys *in segment 6 of domain III [54]. Brengues *et al. *[30] detected *Ile1011Met *within a population of *A. aegypti *from Belém (North region) resistant to pyrethroids and DDT. In electrophysiological studies, this mutation was associated with significantly different levels of nerve sensitivity to both permethrin and lambda cyhalothrin [30]. However, the first countrywide screening for this mutation and its correlation with resistance did not occur until 2009 [17,23]. Martins *et al*. [17] observed that in *A.aegypti *from Natal (Northeast Brazil) the frequency of the resistant allele (*1011Met *= 0.529) was higher in individuals resistant to cypermethrin than in susceptible individuals (0.425), although this difference was not significant. In the present study, we observed a significant association between the *1011Met *frequency and resistance to cypermethrin in the population from Crato (*p *= 0.0242). Martins *et al*. [23] showed that the *1016Ile *allele was found almost exclusively in the Midwest region of Brazil, being absent in the Southeast and Northeast, although no test for association with resistance was performed. In the present study, the *1016Ile *allele was detected for the first time in *A. aegypti *populations from the Northeast of Brazil (Crato and Juazeiro do Norte). We did not detect any association between the *1016Ile *variant and resistance.

The detection of significant association with the resistance phenotype for a *kdr *allele (*1011Met*) shown previously to have a functional effect on nerve response following insecticide exposure [30], and hence with potential importance for the knockdown phenotype, has important implications for control programmes since the continuous use of PY adulticides may promote a drastic increase in frequency in local mosquito populations potentially leading to fixation, as demonstrated by García *et al*. [55] in a study on *A. aegypti *from Mexico. Similarly, Lynd *et al*. [56], observed a rapid increase in *1014Phe *frequency in *An. gambiae *from southern Ghana over a 5-year period. In addition to mutations in the target site of PY, resistance may also have a metabolic basis. Our study detected no significant alterations for MFOs (cytochrome P450s) which have been demonstrated previously to be capable of metabolizing pyrethroids *in vitro *[57]. However, microarray studies of either field samples or laboratory colonies phenotyped for insecticide resistance have previously implicated particular cytochrome P450s in the resistance phenotype [57-59]. Alterations in activity of one, or a few, P450s may not be detectable by the broad MFO assay. However, we did detect significant increase in GST activity and previous studies have shown an association between an increase of GSTs activity and resistance to PY, although some of the populations studied, including those studied here, have also been exposed to OP [12,17,49,60], complicating interpretation.

The status of temephos and cypermethrin resistance in these *A. aegypti *populations reinforces the need for a constant surveillance of mosquito susceptibility against insecticides used in control programs, as well as their effectiveness in the field. This must begin before, or as soon as the insecticide starts to be utilized, such that initial resistance levels are determined, so facilitating resistance management. In general, resistance to chemical insecticides is a multi-factorial trait that may be affected by environmental (availability and types of breeding sites), operational (insecticide application frequency and amount, period of exposure) and genetic (metabolic genes and target-site alteration) factors. Additionally, *A. aegypti *populations from Brazil possess different genetic backgrounds, especially those from the Northeast region [43], a fact that could be behind the diverse resistance mechanisms to the same insecticide developed in different populations. This reinforces the suggestion of Rawlins *et al*. [61], that control strategies must be adjusted to each place according to local peculiarities. A broad program such as PNCD should take this into consideration.

## Competing interests

The authors declare that they have no competing interests.

## Authors' contributions

EPL and MHSP contributed equally to the study. EPL, AES and MOFG conceived the idea of the study; MAVMS designed and coordinated the bioassays, biochemical and semi field tests; UMS, LNO, CNB and CCPN have collected the samples and performed the field-simulated experiments; EPL, APA and EVGS carried out the bioassay and biochemical tests and its analysis; CFJA designed and coordinated the molecular study; EPL and MHSP carried out the molecular assays; CSW and MHSP performed the sequence alignment and statistical analysis; EPL, MHSP, MOFG, MAVMS, CSW and CFJA helped to draft the manuscript and all authors read and approved the final version. MAVMS is guarantor of the paper.
